# Feasibility, acceptability and potential effectiveness of a mobile health (mHealth) weight management programme for New Zealand adults

**DOI:** 10.1186/2052-9538-1-10

**Published:** 2014-07-27

**Authors:** Cliona Ni Mhurchu, Robyn Whittaker, Hayden McRobbie, Kylie Ball, David Crawford, Jo Michie, Yannan Jiang, Ralph Maddison, Wilma Waterlander, Katie Myers

**Affiliations:** National Institute for Health Innovation, University of Auckland, Private Bag 92019, Auckland, New Zealand; Wolfson Institute of Preventive Medicine, Queen Mary University of London, London, United Kingdom; Centre for Physical Activity and Nutrition, Deakin University, Melbourne, Australia

**Keywords:** Obesity, Text message, mHealth

## Abstract

**Background:**

Mobile health (mHealth) behaviour change programmes use mobile phones and the internet to deliver health information and behaviour change support to participants. Such programmes offer a potentially cost-effective way to reach many individuals who do not currently access weight loss services. We developed a mHealth weight management programme using proven face-to-face behaviour change techniques and incorporating target population input. Our aim was to evaluate the feasibility, acceptability and potential effectiveness of this programme for ethnically diverse adults with a view to informing a larger trial.

**Results:**

Fifty three adults who had a BMI of ≥25 kg/m^2^ and wanted to lose weight (81% female, mean age 42 years, mean BMI 35.7 kg/m^2^, 26% Maori, 34% Pacific) received the eight-week mHealth weight loss programme. Anthropometric measures were taken at two face-to-face assessments at baseline and 12-weeks (i.e. four weeks after cessation of intervention).

Twelve-week follow-up measurements were available for 36/53 participants (68%). Non-completers were younger and more likely to be male and of Pacific ethnicity. Thirty five participants (66%) reported reading ‘all or most’ text messages sent and 96% responded to at least one text data collection question over the eight-week active intervention period. Eighty one per cent of participants logged in to the study website at least once during the eight-week study period. In the intention-to-treat analysis, mean weight change was -1.0 kg (SD 3.1) at 12 weeks (p = 0.024) and change in BMI was -0.34 kg/m^2^ (SD 1.1) (p = 0.026). In the completers only analysis (n = 36), mean weight change was -1.4 kg (SD 3.6) (p = 0.023) and change in BMI was -0.50 kg/m^2^ (SD 1.3) (p = 0.025).

**Conclusions:**

A mHealth weight management programme is feasible to deliver to an ethnically diverse population. Changes in body weight and BMI at 12 weeks indicate that the programme could be effective in supporting people with weight loss. However, the high dropout rate indicates a need for further improvements to the programme.

**Trial registration:**

ACTRN12612000850875

## Background

High body mass index (BMI) is one of the leading risk factors contributing to the global burden of disease [1]. It is estimated that a third of the global population is overweight, but there are notable regional differences [2]. Among high income regions, the USA has the greatest age-standardised mean BMI (28.4 kg/m^2^), followed by Australasia (27 6 kg/m^2^). In New Zealand (NZ), almost one in three adults (28%) are obese and a further 35% are overweight [3]. Nationally, significant ethnic and socioeconomic disparities exist; 62% of Pacific and 44% of Maori (indigenous New Zealander) adults are obese compared with 26% of European New Zealanders, whilst those living in deprived areas are 1.6 times more likely to be obese than those living in the least deprived areas [3]. The economic burden of obesity is significant and the combined cost of health care and lost productivity is estimated at NZ$849 million/year [4].

Guidelines for the prevention and management of overweight and obesity generally recommend multi-component interventions, including physical activity and dietary behaviour modification, education and skills training [5–7]. One example is the Weight Action Programme (WAP) [8], a weight loss programme developed in the United Kingdom (UK) for multi-ethnic groups in areas of high deprivation. It is a comprehensive package of cognitive, behavioural and educational interventions including dietary advice, self-monitoring, exercise targets and cue management, delivered in a group-based format by trained facilitators over eight weeks. Whilst such face-to-face approaches to weight management are effective [9], they can be expensive, have limited reach and are unsuitable for those who work or live at a distance from group venues. The broad population penetration of mobile and wireless technologies and advancements in their application offers a potentially cost-effective way to reach many individuals who do not currently access weight loss services. Ninety per cent of NZ households have access to a mobile phone and 75% have access to the internet (63% via broadband) [10].

Mobile health (mHealth) behaviour change programmes use mobile phones and the internet to deliver health information and behaviour change support to participants. The flexible nature of mHealth programmes means they can be tailored to specific age, cultural and socioeconomic groups and messages can be delivered directly to participants at appropriate times wherever they might be. Evidence to date indicates text messaging is effective as a tool to support behaviour change [11] and healthcare via mobile phones can improve health outcomes and care processes across a range of clinical areas [12].

Previous NZ feasibility research demonstrated strong support for a mHealth weight management intervention with 75% of Maori and 65% of non-Maori surveyed saying they would use a mobile-phone based weight loss intervention [13]. We developed a mHealth weight management programme based on the UK WAP programme [8] using proven face-to-face behaviour change techniques and incorporated target population input obtained from focus groups and an online survey (described below). Our aim was to evaluate the feasibility, acceptability and potential effectiveness of this programme for New Zealand adults with a view to informing a larger trial.

## Methods

### Programme development

Our mHealth programme was based on a pre-existing group-based weight management programme developed for delivery in multi-ethnic localities in areas of high deprivation in the UK [8]. We modified the programme for delivery to a NZ population via text messages and the Internet using a formative development process incorporating two phases [14]: (1) expert input by members of the project team (CNM, RW, RM, KB, DC, HMR, KM) on effective behaviour change theory, techniques and programmes and (2) target population input via focus groups (n = 20 participants), one-on-one phone interviews (n = 5), and a quantitative online survey (n = 120) on types of weight loss information desired and preferences for programme features [15]. Consistent with a recent meta-analysis of effective intervention techniques, expert input determined that core components of an evidence-informed programme should include: self-monitoring of behaviour; prompting intention formation; promoting goal-setting; providing feedback on progress; and prompting review of behaviour goals [16]. Input from the target population (people who were overweight and wanted to lose weight) identified that the following programme components were desired: personalisation of messages; immediate and informative text messages; practical information; culturally appropriate language; social support options; and weight tracking functions [15]. In keeping with the objective of developing a programme appropriate for an ethnically diverse population, the online survey had good Maori and Pacific representation (n = 75 participants, 63%), as did (to a lesser extent) the focus groups and interviews (n = 7, 28%) [15].

A 12-week weight management programme (eight-week active intervention and four-week maintenance/follow-up) was developed comprising three modules: text messages, a hard-copy toolkit and a secure participant website [15]. A library of 130 text messages was developed that focused on motivation, goal setting, enlisting social support, behaviour monitoring and encouragement to use other study modules. Participants were sent an average of two texts per day over the eight-week intervention period. Examples included: “*It is important to set small goals each week. The toolkit has suggestions - pick one goal to get started on this week, write it down and go for it!*” and “*Hi [NAME], we are here to support you along the way. Each week we will ask about your step count and goals and you can track your progress online*”. All messages were personalised (i.e. used participants’ names) and tailored according to: (1) whether participants were the main household grocery shopper/cook; and (2) whether they had children or not [15]. There is a significant association between self-monitoring and weight loss [17] so two data collection questions were also sent by text each week asking participants to respond with their most recent daily pedometer step count and to rate their progress in achieving their weekly behaviour change goal on a scale of 1 (not well at all) to 10 (brilliantly).

The toolkit served as a source of more detailed information with material to support personal plans (setting and recording weekly goals); behaviour monitoring (food diaries, step count diaries, weight records); healthy shopping; recipes; energy content of common foods; and case histories of successful weight loss. The website provided a blog to enable participants to share their stories and experiences and graphical displays of daily step counts and progress in meeting behavioural goals. The three modules were designed to be integrated; for example, text messages encouraged participants to set short-term goals, the toolkit provided practical examples of goals, text messages asked about perceived progress in achieving goals, participants responded to these messages and reported progress was displayed visually on website graphs.

### Design and setting

The feasibility study was conducted in Auckland, NZ, between September and December 2012. A 12-week intervention and follow-up period was chosen because it was considered sufficient to assess participant recruitment and retention rates, acceptability of the programme and potential effectiveness based on short-term weight changes. The study protocol and procedures were approved by the Upper South B Regional Ethics Committee on 13 March 2012 (URB/12/EXP/010). Baseline and follow-up assessments took place at the University of Auckland and the intervention was delivered directly to participants via mobile phone and the Internet.

### Study participants and recruitment

Participants were recruited via advertisements placed in local newspapers and local community venues such as libraries, community centres and general practices, email lists of University staff and other relevant organisations and direct mail drops. Adults aged 18 years and older who had a BMI of ≥25 kg/m^2^ and wanted to lose weight were eligible to take part. Participants also needed to have Internet access (at home, work or local library) and own a mobile phone. Individuals who could not read or understand English were ineligible. Volunteers responding to advertisements called the study centre whereby they were given further information on the study procedures and their eligibility to participate was checked. Eligible individuals were given an appointment to attend a baseline assessment at the University of Auckland.

### Study measures

Data collected by self-completed questionnaire at the baseline visit were: demographics (date of birth, sex, ethnicity, educational qualifications, household income, household composition, area of residence); relevant lifestyle and medical history including smoking, alcohol and sleep habits, history of diabetes and cardiovascular disease and methods used to support previous weight loss attempts; mobile phone number (for delivery of the programme); and study referral source (to evaluate success of study recruitment methods). Height and weight were measured by Research Assistants to calculate body mass index (BMI). Height without shoes was measured twice to the nearest 1.0 cm using a Seca model 214 stadiometer and the average was calculated. Weight without shoes in light indoor clothing was measured twice to the nearest 0.1 kg using Salter model 9064 electronic scales and the average was calculated. Since weight loss reduces blood pressure levels, three seated blood pressure (BP) measurements were taken at least three minutes apart using an OMRON model T9P automatic blood pressure monitor. The second and third readings were recorded and the average was calculated. Participants returned for a final follow-up visit at 12 weeks at which body weight, BP, participant feedback on the utility of the mHealth weight loss programme and any recommendations for improvements/modifications were recorded.

### Intervention

Following informed consent and baseline data collection, participants were enrolled to receive the eight-week mHealth weight loss programme. They were loaned pedometers for the duration of the study to enable them to self-monitor their physical activity [17], given food diaries to facilitate dietary monitoring [17] and access to a study website where users could view programme information and review their progress.

### Outcomes

The primary outcomes of interest were feasibility (recruitment and retention rates) and acceptability (frequency of use and participant feedback) of the programme. Frequency of programme use was evaluated using the question “How many of the texts did you read?” (All/Most/About half/A few/None), and data on responses to text message questions and study website log-ons. Secondary outcome measures were changes in BP, body weight and BMI. The latter two measures provided an indication of potential programme effectiveness and would assist with calculating sample size necessary for a larger trial.

### Sample size and data analysis

This was a non-randomised feasibility study so a formal sample size calculation was not undertaken. We aimed to recruit 50 ethnically diverse participants. The sample size of 50 was a pragmatic decision based largely on study resources. Descriptive analyses were undertaken to describe participant recruitment and retention rates, demographics and measured body weight at baseline and 12 weeks. An intention-to-treat (ITT) analysis was conducted where missing data at 12 weeks were imputed using the ‘baseline value carried forward’ approach (i.e. no weight loss). Both a parametric t-test and non-parametric Wilcoxon signed rank test for paired samples (i.e. before and after) was used to evaluate the change in body weight and BMI from baseline to 12 weeks. A ‘completers only’ analysis was also conducted, including only participants who provided follow-up weight data. Participant feedback on the programme was summarised. Statistical analysis was conducted using SAS version 9.3 (SAS Institute Inc. Cary NC). A 5% level of significance (two-sided) was used in all tests.

## Results

### Recruitment and baseline participant characteristics

Study recruitment took place April to September 2012. A total of 154 individuals initially expressed interest in taking part in the feasibility study and 93 were assessed for eligibility. When the target sample size was reached, recruitment was closed and remaining registrants were not screened. Of those screened, six were excluded because they did not meet study eligibility criteria. A further 29 individuals were excluded because they lived outside the Auckland area and could not attend clinic appointments. In total, 58/93 screened individuals met the eligibility criteria and were invited to a baseline appointment but five did not attend, leaving 53 participants who were enrolled into the programme.

Participants were recruited via email lists (n = 22), heard about the study from family, friends or colleagues (n = 15), responded to adverts in local newspapers (n = 13), or became aware of the study through other means (n = 3). On average, participants were 42 years old (age range: 19 to 68) and 81% (n = 43) were female (Table 1). Twenty six per cent self-identified as Maori and 34% as Pacific. Twenty participants (38%) reported their highest educational qualification was a school certificate (equivalent to 12 years’ formal schooling) and most (n = 39) had an annual household income exceeding NZ$50,000. Mean baseline BMI of all participants was 35.7 (SD 6.8) kg/m^2^ and 92% had tried to lose weight at least once previously.

**Table 1:** **Baseline characteristics of study participants**

	All participants	Completers	Non-completers
	***n =***53	(***n =***36)	(***n =***17)
Age, years, mean (SD)	42 (13)	45 (13)	37 (11)
Sex, n (%)			
- Male	10 (19)	5 (14)	5 (29)
- Female	43 (81)	31 (86)	12 (71)
Ethnicity, n (%)*			
- Maori	14 (26)	10 (28)	4 (24)
- Pacific	18 (34)	8 (22)	10 (59)
- New Zealand European	23 (43)	19 (53)	4 (24)
- Other	9 (17)	5 (14)	4 (24)
Highest educational qualification, n (%)			
- School certificate	20 (38)	12 (33)	8 (47)
- University or polytechnic degree or diploma	30 (57)	23 (64)	7 (41)
- Other	3 (6)	1 (3)	2 (12)
Annual household income before tax, n (%)			
- ≤$50,000	11 (21)	8 (22)	3 (18)
- $50,001 to $100,000	25 (47)	16 (44)	9 (53)
- ≥$100,001	14 (26)	10 (28)	4 (23)
- Decline to answer	3 (6)	2 (6)	1 (6)
Body weight, kg, mean (SD)	100.9 (21.3)	99.8 (21.7)	103.3 (20.9)
BMI, kg/m^2^, mean (SD)	35.7 (6.8)	35.4 (7.1)	36.3 (6.3)
Systolic blood pressure, mmHg, mean (SD)	121.8 (16.9)	123.4 (16.2)	118.4 (18.4)
Diastolic blood pressure, mmHg, mean (SD)	75.6 (11.2)	76.0 (11.1)	74.7 (11.6)
Diagnosed co-existing conditions, n (%)*			
- Diabetes	6 (11)	6 (17)	0 (0)
- High blood pressure	10 (19)	8 (22)	2 (12)
- High blood cholesterol	5 (9)	3 (8)	2 (12)
- Coronary heart disease or stroke	2 (4)	2 (6)	0 (0)
- Osteoarthritis	4 (8)	3 (8)	1 (6)
Smoking status, n (%)			
- Current smoker	5 (9)	2 (6)	3 (18)
- Ex-smoker	21 (40)	15 (42)	6 (35)
- Never smoked	27 (51)	19 (53)	8 (47)
Usual alcohol intake, n (%)			
- Never	9 (17)	5 (14)	4 (24)
- Once a week or less	34 (64)	22 (61)	12 (70)
- 2-3 times per week	8 (15)	7 (19)	1 (6)
- 4 times or more per week	2 (4)	2 (6)	0 (0)
Sleep, n (%)			
- Weekday or workday night, hours, mean (SD)	6.6 (1.1)	6.7 (1.0)	6.5 (1.1)
- Weekend or non-work night, hours, mean (SD)	7.3 (1.2)	7.4 (1.1)	7.1 (1.4)
Self-reported knowledge of weight loss and dieting, n (%)			
- None	1 (2)	1 (3)	0 (0)
- Little	12 (23)	8 (22)	4 (24)
- Moderate amount	21 (40)	15 (42)	6 (35)
- A lot	19 (36)	12 (33)	7 (41)
Weight loss history, n (%)			
- Previously tried to lose weight, n (%)	49 (92)	33 (92)	16 (94)
- Number of weight loss attempts in past 5 years			
○ One	10 (20)	8 (24)	2 (13)
○ 2-4	19 (39)	13 (39)	6 (38)
○ 5 or more	18 (37)	11 (33)	7 (44)
- Methods used*			
○ Changing food intake, n (%)	42 (86)	29 (88)	13 (81)
○ Doing more exercise, n (%)	47 (96)	32 (97)	15 (94)
○ Use of laxatives and/or diuretics, n (%)	3 (6)	1 (3)	2 (13)
○ Prescribed weight loss medication, n (%)	5 (10)	4 (12)	1 (6)
○ Over-the-counter weight loss treatment, n (%)	6 (12)	6 (18)	0 (0)

*These questions allowed participants to select more than one option. As such the total number of options reported could sum to more than 100%.

### Study retention

Over the 12-week study period, four participants withdrew from the study and one person was lost to follow-up (could not be contacted). Twelve participants did not attend their 12-week follow-up appointment so final measurements were obtained for 36 participants (68%). Table 1 reports the characteristics of those who did and did not complete study follow-up. Compared with those who completed the study, non-completers were younger and more likely to be male and of Pacific ethnicity. Non-completers were also more likely to report ‘a lot’ of knowledge of weight loss/dieting at baseline and having attempted to lose weight five or more times in recent years.

### Programme adherence (frequency of use of intervention)

Thirty five of the original 53 participants (66%) reported reading ‘all’ or ‘most’ texts sent during the eight-week intervention phase. Fifty one (96%) responded to at least one data collection text question on daily pedometer step counts and/or rated their progress in achieving their weekly behaviour change goal. In total, 210 responses were received to the step count questions (from a possible total of 392, excluding participant withdrawals) and 250 replies to the goal success questions (from a possible total of 392). Response rates declined over time (weeks 1–8) from 77% to 4% (step count) and 79% to 40% (progress towards goal). In addition, participants could ‘text an expert’ during the programme intervention phase and receive a reply to their queries within 24 hours. Nine ‘text an expert’ questions were sent in by six participants and these ranged from requests for quick healthy meal ideas, information on the ideal number of steps per day, to ideas on how to do more exercise.

**Figure 1:**
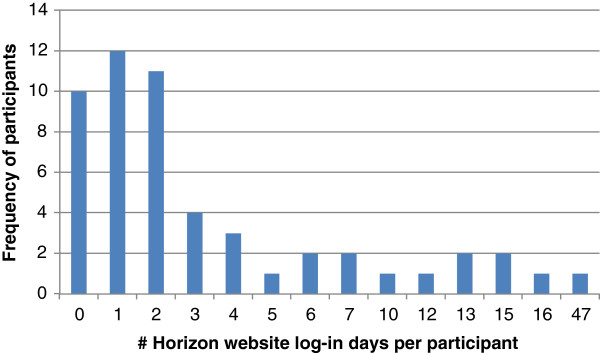
**Website log-in days per participant.**

Forty three participants (81%) logged in to the study website at least once over the total 16-week duration of the study (September to December 2012). Most (70%) logged on three separate days or less and the distribution of usage was skewed with a small number of participants accounting for the highest number of log-in days (Figure 1). The blog was the most popular webpage by far with 1203 total hits (43 individual viewers) followed by the step count graphs (125 hits, 22 viewers), progress towards goals graphs (106 hits, 22 viewers) and the toolkit webpage (86 hits, 39 viewers). Over time, blog page visits declined however, from 212–222 hits/week in weeks 2–5 of the study to 0–2 hits/week in weeks 13–16. There were 75 blog posts in total, comprising 48 entries by 13 study participants and 27 blog responses or posts by six members of the research team. Posts peaked in week 5 (13 posts by 7 individuals) and declined to zero by week 11. A variety of topics were discussed ranging from sugar cravings to enlisting family support. Participants shared success stories and tips as well as difficulties and challenges faced.

### Acceptability of programme and participant feedback

At follow-up participants were asked for feedback about the different aspects of the programme. The most liked features reported spontaneously (via free text) were receiving texts (34%) and recording step counts (21%). When asked directly about specific aspects of the programme, the most liked features were goal setting (43% reported that they found this extremely or very helpful) and recording step counts (45%), while fewer found keeping a food diary or monitoring their weight extremely or very helpful (26% and 17% respectively) (Table 2). With regards to text messages, the characteristics people reported liking most were their motivational aspect (28%) and the clear, practical tips they provided (15%). In contrast, characteristics disliked included the perceived impersonal and generic nature of the messages (26% combined) and repetition (13%). Feedback on the toolkit showed that the sections people liked best were those on the Horizon study team (47% gave a rating of 4 or 5 on a scale of 1 [not at all] to 5 [very much]), goals and tips (45% gave a rating of 4 or 5), and myth busting (40%). Overall, 28% of all study participants (42% of study completers) rated the programme as ‘extremely helpful’ or ‘very helpful’ (Table 2). The remainder either replied neutrally or did not respond to the question. No respondent reported the programme was not at all helpful.

**Table 2:** **Participant feedback on programme**

General	***N***(% of all study participants [n = 53])
Did you set yourself any goals?	
- Yes, every week	10 (19)
- Yes, some weeks	28 (53)
If so, was it helpful?	
- Extremely or very helpful	23 (43)
Did you keep a food diary?	
- Yes, every week	4 (8)
- Yes, some weeks	23 (43)
If so, was it helpful?	
- Extremely or very helpful	14 (26)
Did you record your body weight?	
- Yes, every week	4 (8)
- Yes, some weeks	22 (42)
If so, was it helpful?	
- Extremely or very helpful	9 (17)
Did you record your step counts?	
- Yes, every week	17 (32)
- Yes, some weeks	18 (34)
If so, was it helpful?	
- Extremely or very helpful	24 (45)
**Text messages**	
Was the number of texts received….	
- Too many	16 (30)
- About right	21 (40)
- Not enough	0 (0)
- *No response*	*16 (30)*
What, if anything, did you like about the texts (free text answers)?	
- Motivational	15 (28)
- Clear practical tips	8 (15)
- Provided reminders	4 (8)
- Interactive	4 (8)
- Positive	4 (8)
What, if anything, did you dislike about the texts (free text answers)?	
- Impersonal	8 (15)
- Repetitive	7 (13)
- Generic	6 (11)
- Irrelevant	5 (9)
- Step count question	5 (9)
**Website**	
How helpful did you find the blog?	
- Extremely or very helpful	8 (15)
- Moderately or a little helpful	10 (19)
- Not helpful or not used	3 (6)
- *No response*	*32 (60)*
How helpful did you find the website graphs of step counts and progress towards goals?	
- Extremely or very helpful	10 (19)
- Moderately or a little helpful	5 (9)
- Not helpful or not used	6 (11)
- *No response*	*32 (60)*
Please rate how much you liked the Goals and Tips section in the toolkit on a scale of 1 (not at all) to 5 (very much)	
- 1 or 2	3 (6)
- 3	11 (21)
- 4 or 5	24 (45)
- *No response*	*15 (28)*
Please rate how much you liked the Tips for Healthy Shopping section in the toolkit on a scale of 1 (not at all) to 5 (very much)	
- 1 or 2	15 (28)
- 3	5 (9)
- 4 or 5	14 (26)
- *No response*	*19 (36)*
Please rate how much you liked the Recipes section in the toolkit on a scale of 1 (not at all) to 5 (very much)	
- 1 or 2	9 (17)
- 3	10 (19)
- 4 or 5	7 (13)
- *No response*	*27 (51)*
Please rate how much you liked the Walking Tracks section in the toolkit on a scale of 1 (not at all) to 5 (very much)	
- 1 or 2	14 (26)
- 3	6 (11)
- 4 or 5	6 (11)
- *No response*	*27 (51)*
Please rate how much you liked the Energy Content of Common Foods section in the toolkit on a scale of 1 (not at all) to 5 (very much)	
- 1 or 2	7 (13)
- 3	14 (26)
- 4 or 5	12 (23)
- *No response*	*20 (38)*
Please rate how much you liked the Understanding the Basics section in the toolkit on a scale of 1 (not at all) to 5 (very much)	
- 1 or 2	9 (17)
- 3	8 (15)
- 4 or 5	17 (32)
- *No response*	*19 (36)*
Please rate how much you liked the Getting Support section in the toolkit on a scale of 1 (not at all) to 5 (very much)	
- 1 or 2	15 (28)
- 3	10 (19)
- 4 or 5	10 (19)
- *No response*	*18 (34)*
Please rate how much you liked the Myth Busting section in the toolkit on a scale of 1 (not at all) to 5 (very much)	
- 1 or 2	9 (17)
- 3	8 (15)
- 4 or 5	21 (40)
- *No response*	*15 (28)*
Please rate how much you liked the Horizon Stories section in the toolkit on a scale of 1 (not at all) to 5 (very much)	
- 1 or 2	15 (28)
- 3	3 (6)
- 4 or 5	16 (30)
- *No response*	*19 (36)*
Please rate how much you liked the Horizon Team section in the toolkit on a scale of 1 (not at all) to 5 (very much)	
- 1 or 2	9 (17)
- 3	4 (8)
- 4 or 5	25 (47)
- *No response*	*15 (28)*
**Overall rating of programme with respect to helping develop healthier habits**	
- Extremely or very helpful	15 (28)
- Moderately or a little helpful	6 (11)
- Not helpful	0 (0)
- *No response*	*32 (60)*

### Change in body weight and BMI at 12 weeks

Change in body weight was analysed in two ways. ITT analysis showed a significant change in body weight from baseline (M = 100.9 kg, SD = 21.3) to 12 weeks (M = 99.9 kg, SD = 21.9). Mean weight change was -1.0 kg (SD 3.1) at 12 weeks (paired t-test, p = 0.024) and change in BMI was -0.34 kg/m^2^ (SD 1.1) (p = 0.026) (Table 3). In the completers only analysis (n = 36), there was a significant change in body weight from baseline (M = 100.9 kg, SD = 21.3) to 12 weeks (M = 98.3 kg, SD = 22.5). Mean weight change was -1.4 kg (SD 3.6) at 12 weeks (paired t-test, p = 0.023) and change in BMI was -0.50 kg/m^2^ (SD 1.3) (p = 0.025). Five participants lost 5% or more of their baseline weight. There were no significant changes in systolic (p = 0.796) or diastolic (p = 0.919) blood pressure.

**Table 3:** **Change in body weight and BMI at 12-weeks**

	Intention to treat analysis				Completers only analysis			
	***n =***53				***n =***36			
	Mean (SD)				Mean (SD)			
	Baseline	12 weeks	Change from baseline	p-value*	Baseline	12 weeks	Change from baseline	p-value*
Body weight (kg)	100.9 (21.3)	99.9 (21.9)	-1.0 (3.1)	0.024	100.9 (21.3)	98.3 (22.5)	-1.4 (3.6)	0.023
BMI (kg/m^2^)	35.7 (6.8)	35.4 (7.1)	-0.3 (1.1)	0.026	35.7 (6.8)	34.9 (7.4)	-0.5 (1.3)	0.025
	**Median (range)**				**Median (range)**			
	**Baseline**	**12 weeks**	**Change from baseline**	**p-value**	**Baseline**	**12 weeks**	**Change from baseline**	**p-value**
Body weight (kg)	97.9 (59.8, 165.0)	92.0 (60.4, 163.7)	0.0 (-13.1, 3.7)	0.066	97.9 (59.8, 165.0)	91.0 (60.4, 163.7)	-0.6 (-13.1, 3.65)	0.066
BMI (kg/m^2^)	34.6 (26.4, 56.8)	34.1 (25.4, 56.9)	0.0 (-4.6, 1.5)	0.07	34.6 (26.4, 56.8)	34.0 (25.4, 56.9)	-0.2 (-4.6, 1.5)	0.070

*Paired t-test on the mean (parametric).

Wilcoxon signed rank test on the median (non-parametric).

## Discussion

This feasibility study has shown that a weight management programme delivered by mobile phone and the Internet is feasible to deliver to ethnically diverse New Zealand adults. Changes in body weight and BMI at the 12-week follow-up visit indicate that the programme could be effective in supporting people with weight loss. However, the high dropout rate, particularly amongst young, male and Pacific participants indicates a need for further improvement.

We recruited 53 participants within a relatively short timeframe (approximately six weeks). As is common in weight loss studies, the majority were women (81%). In contrast to many studies however [18, 19] our sample was ethnically diverse (60% of participants were of Maori or Pacific ethnicity), demonstrating both a demand for weight management programmes amongst these populations and their ready acceptance of technologies such as mobile phones and the Internet. Advertising using email lists and networks/word-of-mouth were the most successful recruitment strategies.

The short duration of this feasibility study was a limitation. A longer intervention period might have produced greater effects on weight loss. However, it is also possible that a longer study might have experienced greater loss to follow-up. Thirty two per cent of participants withdrew from the study or did not return for their follow-up visit at 12 weeks. Attrition is a serious problem because of its potential to bias results and reduce the validity of study findings [20]. Systematic reviews of weight loss trials with follow-up periods ranging from 12 weeks to two years report loss to follow-up rates of 20-60% [21, 22], and a review of text message weight loss interventions where 11/14 studies had follow-up periods of 12 weeks or less found almost half the studies had loss to follow-up rates exceeding 20% [23].A recent six-month pilot of a smartphone application for weight loss reported a 38% attrition rate [24]. Non-completers in our study were more likely to be young, male and of Pacific ethnicity, which may indicate that the study intervention did not engage these groups sufficiently. Given the risk of bias associated with study attrition, attention should be focussed on ways to increase participant retention rates in future mHealth interventions and randomised controlled trials. Increased social support provided via social media sites may be useful in this regard [25].

Although behavioural aspects of the programme (e.g. goal-setting and recording food intake) known to be consistent with best practice for behaviour change interventions [16] were well-received, not many participants reported using them weekly. Self-monitoring is important because it has been linked consistently to successful weight loss [17]. Similarly, responses to text questions and website usage were low and declined significantly over time. These aspects of the programme were optional and participants were not required to respond by text or use the study website. However, it is possible that the programme was not sufficiently engaging or did not vary often enough to maintain participant commitment. In general, participants reported that they liked receiving text messages; they found them motivational and liked their clear practical tips and reminders for behaviour change. However, a number of people reported that they found the text messages impersonal/generic or repetitive. Therefore, programme effectiveness could potentially be improved further if content and messages were tailored more precisely to the personal characteristics and circumstances of participants.

This was a non-randomised feasibility study that was not statistically powered to detect a difference in weight change over time. However it provides data on potential effectiveness. On average, study participants achieved a mean weight loss of -1 kg (SD 3.1) after 12 weeks and completers had a mean weight loss of -1.4 kg (SD 3.6). This is less than that reported in a large meta-analysis of clinical trials of weight loss interventions ranging from simple advice through to meal replacements and weight loss medications (mean weight loss of 5 to 8.5 kg at 6 months) [26]. It is however similar to that described in a meta-analysis of web-based weight loss interventions, which reported a mean weight loss of 2.2 kg at 12 months [22]. Whilst a review of text messaging interventions reported mixed effects on BMI and weight [23], individual studies reported significantly greater weight loss in intervention compared to control groups ranging from 1.97 kg at 16 weeks [27] to 3.4 kg at 12 months [28]. As such, our short-term results suggest potential effects similar to those achieved by other programmes delivered via the internet or mobile phone.

The programme tested in this study involves minimal contact and is thus more scalable and likely more cost-effective than high intensity face-to-face programmes. Whilst clinical weight loss interventions may produce greater weight loss [26] they are costly and not accessible or appealing to everyone who wishes to lose weight. This non-invasive evidence-based programme offers a further wide-reaching treatment option that could be used alone or as an adjunct to other weight management interventions such as medication, dietary advice or commercial weight loss programmes. Modelling studies suggest that even interventions with modest effects on mean population BMI could still have a major impact on population health and mortality rates [29, 30]. A further strength is that our study included an ethnically diverse sample and input was sought from priority ethnic groups in developing the programme to ensure cultural appropriateness. Feedback from study participants suggests the programme had broad appeal. However, the study sample was highly educated and had high household incomes relative to the average New Zealand household. The high programme attrition rate generally and particularly amongst younger, male and Pacific participants, indicates the need for further enhancements to improve engagement and encourage retention. Feedback indicated that a number of participants found the programme messages impersonal and generic. Greater tailoring of messages to individual participant needs and lifestyles might therefore increase participant engagement and retention. It is also possible that the functionality offered by smartphones could enhance the effectiveness of this programme by offering additional features to increase user engagement such as social support [25] and detailed self-monitoring that offers opportunity for real-time feedback [24].

A key aim of this research was to inform the design of a larger future trial. Based on the data collected in this 12-week feasibility study, we estimate that a future trial would require a minimum sample size of approximately 400 (200 per group). This sample size would provide 90% power at 5% level of significance (two-sided) to detect a 1 kg difference in body weight between the intervention group and the control group, assuming a standard deviation of 3.1 (Table 3).The sample size may however need to be increased if significant loss to follow-up is anticipated. It should also be increased to provide sufficient power to undertake important subgroup analyses e.g. effects by ethnic group.

## Conclusions

This study demonstrated the feasibility of delivering a mHealth weight management programme for overweight adults in New Zealand. Regular use of the programme and largely positive feedback indicates that the programme was acceptable to the study population. Recruitment rates were good and the effects of the programme on weight loss at 12 weeks are promising. Further enhancements are however necessary to improve and sustain participant engagement.

## Abbreviations

mHealth: Mobile health
NZ: New Zealand
WAP: Weight action programme
BMI: Body mass index
BP: Blood pressure
ITT: Intention-to-treat
SD: Standard deviation.
